# rMyCoPortal - an R package to interface with the Mycology Collections Portal

**DOI:** 10.3897/BDJ.7.e31511

**Published:** 2019-01-14

**Authors:** Franz-Sebastian Krah, Scott T. Bates, Andrew N. Miller

**Affiliations:** 1 Bavarian Forest National Park, Grafenau, Germany Bavarian Forest National Park Grafenau Germany; 2 Technical University of Munich, Freising, Germany Technical University of Munich Freising Germany; 3 Purdue University Northwest, Westville, United States of America Purdue University Northwest Westville United States of America; 4 University of Illinois Urbana-Champaign, Champaign, United States of America University of Illinois Urbana-Champaign Champaign United States of America

**Keywords:** data portal, database, fungaria, fungi, georeferencing, natural history collections, specimens, MyCoPortal, Symbiota, R package

## Abstract

The understanding of the biodiversity and biogeographical distribution of fungi is still limited. The small number of online databases and the large effort required to access existing data have prevented their use in research articles. The Mycology Collections Portal was established in 2012 to help alleviate these issues and currently serves data online for over 4.3 million fungal records. However, the current process for accessing the data through the web interface is manual, therefore slow, and precludes the extensive use of the existing datasets. Here we introduce the software package rMyCoPortal, which allows users rapid, automated access to the data. rMyCoPortal makes data readily available for further computations and analyses in the open source statistical programming environment R. We will demonstrate the core functions of the package, and how rMyCoPortal can be employed to obtain fungal data that can be used to address basic research questions. rMyCoPortal is a free and open-source R package, available via GitHub.

## Introduction

Global climate and land-use change are major threats to life on earth, and studies continue to document how animal and plant distributions and phenology have changed due to these factors (Parmesan 2006). Although many studies exist that report range and phenological shifts in plant and animals, less is known about inter- and intra-annual shifts of fungal fruiting and occurrence. Large-scale fungal phenology patterns have only recently been addressed (Boddy et al. 2014). In the last decade, several long-term observational data in Europe (Gange et al. 2007, Kauserud et al. 2012, Kauserud et al. 2008, Büntgen et al. 2012, Büntgen et al. 2013, Büntgen et al. 2015, Andrew et al. 2018b, Kauserud et al. 2010) and rarely in North America (Diez et al. 2013) were used to study the effects of climate change on fungal fruiting phenology. The majority of studies were based on herbarium, museum and citizen science fruit body observations of mushroom-forming fungi (reviewed in Boddy et al. 2014). Several studies at different spatial scales found shifts of the average fruiting date later in the year and the fruiting season has expanded in both directions (Kauserud et al. 2012, Kauserud et al. 2008, Büntgen et al. 2012, Büntgen et al. 2013, Büntgen et al. 2015, Boddy et al. 2014, Andrew et al. 2018). However, such phenological shifts have been mainly studied in Europe and emerging species distribution models (SDMs) based on future climate projections have yet to be studied.

Open-source data provide an important resource for studying fungal biodiversity (Andrew et al. 2017, Andrew et al. 2018a). The Mycology Collections data Portal (MyCoPortal 2018) has made great efforts to compile fungal specimen metadata that document distributions of fungi, with 42% of the records therein being georeferenced (Table 1, Miller and Bates 2017). The MyCoPortal is built on the open-source Symbiota platform (Gries et al. 2014), and currently serves over 4.3 million unique fungal records, these being primarily specimen-based (Miller and Bates 2017). The 96 specimen-based collections on the MyCoPortal consist of 70 live collections, which maintain their collections data directly on the MyCoPortal, and 26 snapshot collections, which maintain their collections data in their institutional database (i.e., Specify, Emu, Excel, etc.) and update the data on the MyCoPortal periodically. Data is typically updated at least annually without affecting usability. Further, the MyCoPortal is the only worldwide data aggregator for specimen-based fungal records and contains many records not currently present in the iDigBio portal (https://www.idigbio.org) or GBIF (GBIF 2018). Symbiota software contains a GBIF publishing tool that will soon be available in the MyCoPortal to facilitate uploading specimen-based institutional data.

Although the MyCoPortal has been widely used and highly cited (>43 citations since 2015), data from this portal, however, can be difficult to access for automated analyses as the platform requires users to manually download data through a web interface. This procedure is very time consuming, especially when working with complex queries and building large datasets. After download, the data then needs to be further processed before basic exploration can be undertaken.

To this end, the first author has developed software that allows rapid and automated access to a large global database of fungal distribution records, eliminating the need to use the existing web interface. rMyCoPortal is written as a package for the popular R open-source statistical software (R Core Team 2012) to make data contained in MyCoPortal accessible in the R programming environment. In this publication we introduce the core functionality of rMyCoPortal and briefly demonstrate the potential uses of the data. Therefore, we wish to highlight three important data analysis techniques that are facilitated by rMyCoPortal: 1) Visualization of fungal species ranges; 2) creation of heatmaps of fungal biodiversity throughout the world; and 3) modelling of habitat suitability.

## Installation

The package can be downloaded and installed using the R package devtools (Wickham et al. 2018), using the following code:

## Install R package rMyCoPortal

install.packages('devtools')

devtools::install.github('FranzKrah/rMyCoPortal')

library('rMyCoPortal')

# Now Docker needs to be installed.

The download and usage of the package does not require a GitHub account. An account is, however, required if the user would like to actively contribute to functions of rMyCoPortal or launch an issue.

## Usage

### Core package function

Here, we present the core functions of the rMyCoPortal package. rMyCoPortal makes use of several R packages that allow interaction with web data content, including RSelenium (Harrison 2018), XML (Lang and CRAN Team 2018), and httr (Wickham 2017); but also plotting packages such as ggplot2 (Wickham 2016). Selenium allows to access data from website with dynamic content, such as PHP with SQL database. MyCoPortal contains such a database. Selenium provides a suit of routines for web browser automation, e.g., simulating web site queries. Selenium therfore builds a Selenium server within which the web browser is run. Docker containers (https://docs.docker.com) are the recommended way of running a Selenium server (Harrison 2018, vignette "basics" in RSelenium). Containers are packages of code that run quickly and reliably on various computing environment to another and standardise the build across operating systems. Lastly, rMyCoPortal also provides the class ‘records’, introduced to provide user-friendly interaction with the downstream data analysis (e.g., plotting).

### Querying the database

At the core of rMyCoPortal is the function *mycoportal*. Using *mycoportal*, queries can be made to find all records of a known fungal species. Further, all input specifications (i.e., query modifiers) that are present on the website can be adjusted within said function. Some important modifiers are the inclusion of synonyms or the geographic area. The user may also input a higher taxon, e.g., genus or family. The records are then stored in an S4 class object, which can be directly subjected to a variety of plotting functions. The functions *plot_distmap* and *plot_datamap* can be used to visualize species distributions and heatmaps of species diversity, respectively (Fig. 1). The downloaded data records can then further be used for subsequent statistical analysis, for example climate suitability modelling (Fig. 1, R code in vignette in the R package), changes in phenology or to create species lists for a given locality.

The following code demonstrates the usage of basic functions of the rMyCoPortal package:

## Download data for *Amanita muscaria*

am.rec <- mycoportal(taxon = 'Amanita muscaria')

## Plot species distribution

plot_distmap(x = am. rec , mapdatabase= 'state', interactive = FALSE)

## Plot records heatmap for states of USA

plot_datamap(x = am. rec, mapdatabase = 'state', index = 'rec')

### Species distribution modelling

The above code demonstrates the core functionality of the rMyCoPortal package for querying fungal records and also for basic data exploration. Using the *mycoportal* function, we queried the database for all observations for the mushroom-forming fungus *Amanita muscaria* (fly agaric) and modelled the current and future projected habitat suitability using the biomod2 R package (Fig. 1, Georges and Thuiller 2012). Detailed code to create the species distribution models (SDMs) is provided in the vignettes in rMyCoPortal.

## Data limitations

The data contained in the MyCoPortal database is an important resource to address ecological research questions, however there are some limitations to be considered. First, the majority of the data within the database is localized within North America. Second, currently 42% of the records are georeferenced with longitude/latitude values (Table 1), which limits their application. However, most observations are georeferenced to the county level, which allows further localization e.g., via U.S. Gazetteer files. Third, the metadata for each specimen is limited and only provides basic information such as the collector and collection date or the location. For example, the host plant species is often not available (30% of the data has host information) within the metadata. However, information on the host are often available via the herbarium labels, which are available for many specimens in the MyCoPortal. rMyCoPortal implements the function *details*, which can be used to download herbarium labels as images. Further information, such as the host, could then be retrieved manually from the herbarium labels.

## Developer's note

In this paper, we have shown how the R package rMyCoPortal can be utilized to access the Mycology Collections data Portal. This package allows for easy and rapid access to MyCoPortal fungal data, speeding up a process that would otherwise be tedious and slow. Connecting the MyCoPortal database to the R statistical interface opens a wide range of research possibilities, where queried data can be efficiently processed and used to address scientific questions. Further, the rMyCoPortal package has the potential to be modified to access other data portals, such as those for vascular plants (http://swbiodiversity.org/seinet/) or arthropods (http://symbiota4.acis.ufl.edu/scan/portal/), which are also built on the Symbiota platform. We hope this R package inspires scientists to conduct studies related to how fungal biodiversity and biogeography responds to global climate change.

## Web location (URLs) and repository

The package, together with documentation and vignettes, is available on GitHub: https://github.com/FranzKrah/rMyCoPortal

## Usage rights

It is open-source software (published under the GPL public licence, ver. 3).

## Figures and Tables

**Figure 1a.**
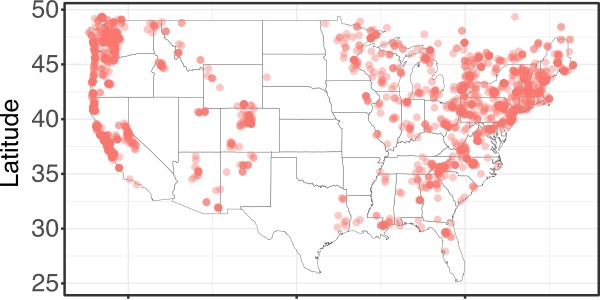
A map made with georeferenced records available from the MyCoPortal showing the species distribution of *Amanita muscaria* throughout the lower 48 contiguous states (using function *plot_distmap*)

**Figure 1b.**
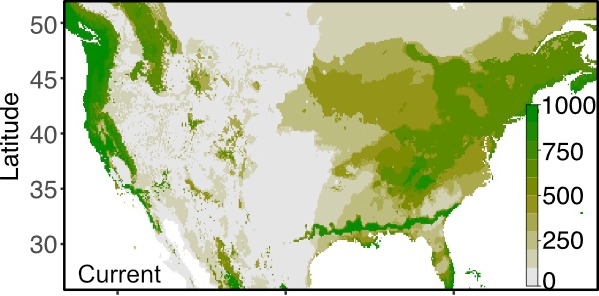
Projected habitat suitability given the current climate based on WorldClim climate data.

**Figure 1c.**
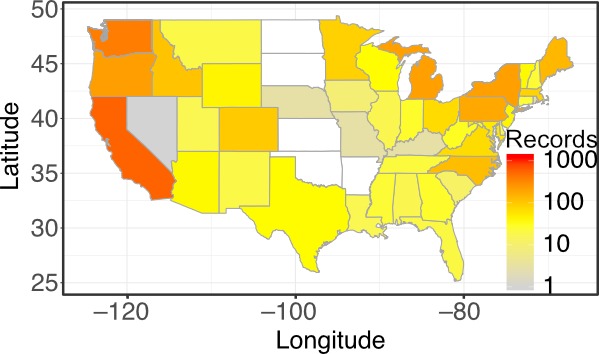
A heatmap showing the number of records of *Amanita muscaria* for the lower 48 contiguous states (using function *plot_datamap*). This function can also be used to plot the number of species if higher taxa are queried.

**Figure 1d.**
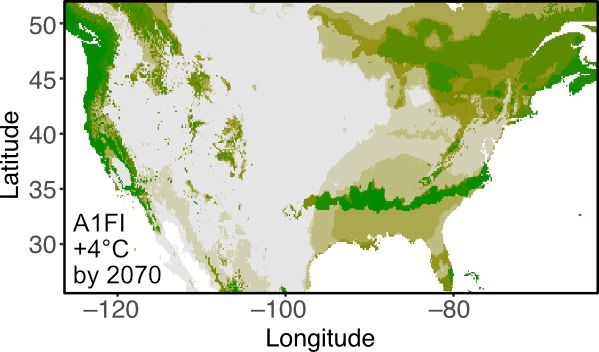
Projected habitat suitability based on future climate, as predicted by the global climate change model CCSM4 and the A1F1 scenario which predicts an increase of 4°C at the end of the century (R code provided in the vignettes of the R package).

**Table 1.** Metadata statistics of the Mycology Collections data Portal (MyCoPortal), retrieved in November 2018 via http://mycoportal.org/portal/collections/misc/collstats.php. The MyCoPortal compiles fungal specimen metadata that document distributions of fungi.

**Collection Statistic**	**Number**
Occurrence records	4,369,313
Georeferenced	1,843,633 (42%)
Imaged	1,913,838 (44%)
Identified to species	3,302,781 (76%)
Families	1,693
Genera	8,314
Species	113,811
Total taxa (including subsp. and var.)	120,275
